# Immunity against HIV/AIDS, Malaria, and Tuberculosis during Co-Infections with Neglected Infectious Diseases: Recommendations for the European Union Research Priorities

**DOI:** 10.1371/journal.pntd.0000255

**Published:** 2008-06-25

**Authors:** Diana Boraschi, Markos Abebe Alemayehu, Abraham Aseffa, Francesca Chiodi, John Chisi, Gianfranco Del Prete, T. Mark Doherty, Ibrahim Elhassan, Howard Engers, Ben Gyan, Ali M. Harandi, Thomas Kariuki, Fred Kironde, Bourema Kouriba, Jean Langhorne, Tamás Laskay, Donata Medaglini, Ole Olesen, Philip Onyebujoh, Carla Palma, Robert Sauerwein, Elopy Sibanda, Ulrich Steinhoff, Aldo Tagliabue, Andreas Thiel, Mahnaz Vahedi, Marita Troye-Blomberg

**Affiliations:** 1 CNR, Pisa, Italy; 2 AHRI-ALERT, Addis Ababa, Ethiopia; 3 Karolinska Institutet, Stockholm, Sweden; 4 University of Malawi College of Medicine, Blantyre, Malawi; 5 University of Florence, Florence, Italy; 6 Statens Serum Institute, Copenhagen, Denmark; 7 University of Khartoum, Khartoum, Sudan; 8 Noguchi Memorial Institute for Medical Research, Legon, Ghana; 9 University of Göteborg, Göteborg, Sweden; 10 Institute of Primate Research, Nairobi, Kenya; 11 University of Makerere, Kampala, Uganda; 12 University of Mali, Bamako, Mali; 13 National Institute of Medical Research, London, United Kingdom; 14 University of Lübeck, Lübeck, Germany; 15 University of Siena, Siena, Italy; 16 EU Commission, Bruxelles, Belgium; 17 WHO, Geneva, Switzerland; 18 ISS, Rome, Italy; 19 Radboud University Nijmegen Medical Center, Nijmegen, The Netherlands; 20 University of Zimbabwe, Harare, Zimbabwe; 21 MPIIB, Berlin, Germany; 22 ALTA, Siena, Italy; 23 Deutsches Rheuma Forschungs Zentrum, Berlin, Germany; 24 WHO, Geneva, Switzerland; 25 Stockholm University, Stockholm, Sweden; New York Blood Center, United States of America

## Abstract

Infectious diseases remain a major health and socioeconomic problem in many low-income countries, particularly in sub-Saharan Africa. For many years, the three most devastating diseases, HIV/AIDS, malaria, and tuberculosis (TB) have received most of the world's attention. However, in rural and impoverished urban areas, a number of infectious diseases remain neglected and cause massive suffering. It has been calculated that a group of 13 neglected infectious diseases affects over one billion people, corresponding to a sixth of the world's population. These diseases include infections with different types of worms and parasites, cholera, and sleeping sickness, and can cause significant mortality and severe disabilities in low-income countries. For most of these diseases, vaccines are either not available, poorly effective, or too expensive. Moreover, these neglected diseases often occur in individuals who are also affected by HIV/AIDS, malaria, or TB, making the problem even more serious and indicating that co-infections are the rule rather than the exception in many geographical areas. To address the importance of combating co-infections, scientists from 14 different countries in Africa and Europe met in Addis Ababa, Ethiopia, on September 9–11, 2007. The message coming from these scientists is that the only possibility for winning the fight against infections in low-income countries is by studying, in the most global way possible, the complex interaction between different infections and conditions of malnourishment. The new scientific and technical tools of the post-genomic era can allow us to reach this goal. However, a concomitant effort in improving education and social conditions will be needed to make the scientific findings effective.

## Background and Rationale

Infectious diseases remain a major health and socioeconomic problem in many low-income countries, particularly in sub-Saharan Africa. Most of the public attention has so far been devoted to the three most devastating diseases, HIV/AIDS, malaria, and tuberculosis (TB). However, in rural and impoverished urban areas of low-income countries, a number of neglected infectious diseases (NIDs) cause massive suffering, although they receive little or no scientific or mass-media attention [1]. By considering all NIDs together, it is obvious that they threaten the health of the poorest to a similar extent as the three major killers [2]–[4]. It has been calculated that a group of 13 NIDs, including Buruli ulcer (*Mycobacterium ulcerae*), cholera (*Vibrio cholerae*), cysticercosis, dracunculiasis (Guinea worm), trematodal infections, hydatidosis, leishmaniasis, lymphatic filariasis (elephantiasis), onchocerciasis (river blindness), schistosomiasis, helminthiasis, trachoma (*Chlamidia trachomatis*), and trypanosomiasis (African sleeping sickness, Chagas disease), affect over one billion people (corresponding to a sixth of the world's population) [4]. For most of these diseases, vaccines are either unavailable, ineffective, or too expensive. Moreover, NIDs often occur in individuals that are also affected by HIV/AIDS, malaria, or TB, indicating that co-infections are the rule rather than the exception in many geographical areas [2]–[5]. In order to develop effective vaccination and treatment strategies, it is essential to understand how protective immunity to a pathogen can be achieved in individuals co-infected with multiple pathogens.

Amongst the numerous specific research programmes launched by several national and international organisations to understand and confront the burden of HIV/AIDS, malaria, and TB, little has been done to specifically address the complex issue of immunity during co-infections with the three major killers and NIDs. So far, it is mainly the European Commission (EC) that has recognised the need to pursue an active research policy for developing new or improved prophylactics and treatments for infectious diseases, including new vaccines and drugs for NIDs, keeping this research area alive in the face of declining national support. While the 6th Framework Programme (FP6) of the EC mainly addressed translational research for HIV/AIDS, malaria, and TB, the new 7th Framework Programme (FP7, 2007–2013) will also include NIDs [6]. The new commitment to NIDs in FP7 creates an unprecedented opportunity to actively address the scientific challenges associated with co-infections between HIV/AIDS, malaria, TB, and NIDs. In addition, the Special Programme for Research and Training in Tropical Diseases of the World Health Organization (WHO/TDR) has shown renewed interest for translational research in NIDs. The recently updated strategy of WHO/TDR aims to support research on neglected needs by fostering innovation for product development, and for access to interventions [7]. It is hoped that industries involved in vaccine and drug development will acknowledge the new strategies against NIDs of the European Union (EU) and WHO/TDR, and follow by implementing research and development activities against NIDs, in particular in the area of immunological correlates of protection and vaccine development.

To address the increasing importance of co-infections, scientists from 14 different countries in Africa and Europe met in Addis Ababa, Ethiopia, on September 9–11, 2007, to identify and prioritise research gaps in this area. The meeting was convened by two ongoing EC-funded initiatives, namely the MUVAPRED integrated project and the BIOMALPAR network of excellence, and gathered high-level scientists, clinicians, and industrial expertise, as well as representatives from the EC and WHO/TDR. This report summarises the consensus of the expert group, which took the name of AFRIEND (African-European Partnership for Neglected Infectious Diseases). It is envisaged that this document could foster a debate in the scientific community and provide recommendations on future actions by the EC and WHO/TDR in the area of co-infections and NIDs.

## Objectives

It is of key importance to focus future research on a detailed understanding of the mechanisms of immunity to pathogens during co-infections between HIV/AIDS, malaria, and TB, and NIDs. This information will be highly relevant for the development of new preventive and therapeutic interventions for use in impoverished areas of disease-endemic countries. Indeed, multiple infections, nutritional status, and level of exposure to microbial/parasitic compounds can alter the reactivity of the immune system in such a way that vaccines may need new/novel formulations. Research priorities should focus on immunological studies in humans, preceded and supported by experimentation on suitable animal models, and should include the following key areas.

### Correlates of Protection

Identification of correlates of protection is of major importance for the development of new effective preventive/therapeutic strategies. Although some progress has been made to identify correlates of protection, much more intensive funding and research are needed to tackle this complex and central area of investigation. Indeed, to date, no convincing correlates of protection have been yet identified for HIV/AIDS, malaria, TB, or NIDs.

As a general assumption, the main correlates of protection in human infections are represented by the presence of pathogen-specific effector cells/molecules (CD8^+^ and CD4^+^ T cells, antibody-producing B cells, neutralising antibodies). More recently, it has become clear that early triggering of innate immune mechanisms can also represent an important determinant of protective immunity. Assays to measure these immune responses are expensive and technically demanding. Therefore, in the search for reliable correlates of protection, priority should be given to the development of simplified, standardised, and low-cost assays.

### Mechanisms of Infection and Immunity at Local Sites

The pathogens responsible for HIV/AIDS, TB, and many of the NIDs invade the human hosts at mucosal surfaces, which act as a primary antimicrobial barrier through non-specific and specific defence mechanisms. In most cases, little attention has been focused on the role of local defences in the control of NIDs. Therefore, the design of strategies to target the local immune defences and to elicit an early mucosal immune response will be crucial.

### Immunological Memory

Immunity to HIV/AIDS, malaria, TB, and NIDs appears to be short-lived, possibly due to the impairment of memory B and T cells and of long-lived plasma cells.

Only a few well-designed studies in humans are available that detail the effects of co-infection on the immune response, but these indicate that important interactions indeed take place that affect the immune response to each of the infecting organisms. For example, HIV infection in primigravid women significantly reduces antibody responses to several important malaria antigens [8]. It is therefore crucial to understand the effects of multiple infections on adaptive immunity and the establishment of immunological memory to the individual pathogens.

Thus, efforts against HIV/AIDS, malaria, TB, and NIDs should include studies to characterise the profile of the memory responses in naturally exposed populations, as well as development of assays to measure memory phenotype and function.

### Impact of Co-Infection on the Outcome of HIV/AIDS, Malaria, TB, and NIDs

More than one billion people worldwide are infected with helminths. Such infections have been shown to cause a range of effects on immune response, characterised by enhanced T helper (Th) 2-type cytokine profile, upregulated regulatory T cell activity, and chronic immune activation. All of these are factors that may have adverse effects on the outcome of subsequent infections and vaccinations. In support of this, studies conducted in animals and humans living in worm-endemic areas have shown that helminths impair resistance against a number of infections, including HIV/AIDS, malaria, and TB [9]–[11]. Accordingly, mortality is high in HIV-positive patients suffering from visceral leishmaniasis, while leprosy seems to be unmasked in co-infected patients when HIV immunosuppression improves with HAART. Infection with schistosomes makes people more susceptible to HIV infection by interfering with immune responses or by increasing the risk of transmission [10],[12]. The interaction between worms and malaria is extremely complex, as people suffering from worm infection and malaria can have higher incidence but reduced severity, while transmission is apparently increased [10],[11]. In sub-Saharan Africa, over 75% of cases of TB are HIV-associated [12]. TB is the leading cause of AIDS-related deaths in low-income countries, and it has been shown that HIV infection increases the risk of progression of TB infection, reactivation of latent infection, and the fatality rate. Other examples of interactions between infections include the interaction between Epstein–Barr virus and malaria, which has been known for many years [13], and HIV and herpes simplex viruses [14]. However, the degree to which this balance is perfected, and the mechanisms by which this is achieved, is far from clear. To understand these complex interactions better, well-designed, controlled intervention studies are needed that could allow us to clarify the mechanisms of protection and to design effective prophylactic and/or therapeutic strategies. Research in this direction is therefore of key importance and should be strongly supported.

### Impact of Non-Infectious Agents on the Outcome of HIV/AIDS, Malaria, TB, and NIDs

Infections in poor communities occur in a setting where the population is exposed to several environmental and social conditions that could influence the immune response to pathogens. These include chronic hunger, micronutrient deficiency, multiple pregnancies, etc. Although the relationship between nutrition and immunity is complex, it is clearly established that nutrient deficiencies can severely impair the immune response. Studies in animal models show an association between malnourishment and disseminated disease development after *Leishmania* infection. However, the complexity of the issue is underlined by the notion that iron deficiency and malnourishment can protect children against severe malaria [15]. Therefore, it becomes essential to investigate and document the basal immunological parameters in a given population, and to determine the “normal values” or reference ranges for the particular population in the endemic set up.

### Host Genetics and Outcome of HIV/AIDS, Malaria, TB, and NIDs

The role of genetic differences in the susceptibility of African populations to HIV/AIDS, malaria, TB, and NIDs has not yet been extensively studied. The significance of host genetic background in disease outcome can be exemplified by the well-known protective effect of the sickle cell trait against malaria in Africa. A number of reports have demonstrated the role of chemokine receptor variants in preventing, delaying, or accelerating progression of HIV infection to AIDS [16]. Similarly, host genetic factors such as HLA haplotype and cytokines/receptors gene polymorphisms have been described to influence susceptibility to both TB and malaria. Recently, a polymorphism in the TLR4 gene has been implicated in protection against malaria [17],[18], while a variant of the TLR2-4 adapter Mal/TIRAP can provide protection against invasive pneumococcal infection, malaria, and TB [19]. It will therefore be important to include genetic markers when elucidating the mechanisms of disease.

### Novel Adjuvants and Their Modes of Action, and Novel Vaccine Formulations

In view of the fact that reactivity of the immune system can be altered in circumstances of multiple infections, malnutrition, and other conditions, novel vaccine formulations able to stimulate protective immunity in states of altered responsiveness should be considered. The development of safe, potent vaccine adjuvants that enhance and direct vaccine-specific immunity is a crucial issue for all new vaccines for human use. Recent advances in immunological research, especially with regard to innate immunity, has resulted in a number of potent adjuvant candidates that can modulate immune responses in a more controlled and specific manner [20]. However, adjuvants suitable for the developed world might not give the same result in populations already exposed to a number of different NIDs. Thus, development of new adjuvants able to promote broad and sustained immune responses at systemic and mucosal levels still remains a major challenge for vaccinology, especially for people living in developing countries.

## Conclusions and Perspectives

Additional research is necessary to understand the mechanisms of immune protection and memory during co-infection with HIV/AIDS, malaria, TB, and NIDs. The AFRIEND consensus meeting identified seven key areas with research gaps, where more attention is needed:

- Correlates of protection
- Mechanisms of infection and immunity at local sites
- Immunological memory
- Impact of co-infection on the outcome of HIV/AIDS, malaria, TB, and NIDs
- Impact of non-infectious agents on the outcome of HIV/AIDS, malaria, TB, and NIDs
- Host genetics and the outcome of HIV/AIDS, TB, malaria, and NIDs
- Novel adjuvants and their modes of action, and novel vaccine formulations

This list is not in order of priority, as all of these issues should be tackled concomitantly, in order to obtain effective outcomes. Global coordination and harmonisation of efforts therefore are of key importance.

To achieve these goals it will be essential to put in place a sustainable network between researchers in disease-endemic countries and researchers in the developed world, which together will implement an integrated immunological research effort across disciplines and diseases. This needs to include promotion of high-level training pathways for African researchers and sustaining their careers in African institutions. All of this will require consensus and support both at the political and social level in low-income countries.

In fact, no scientific achievement, however relevant, could succeed in being applied without a proper context of social acceptability, feasibility, and affordability in low-income countries. Thus, research and development of vaccines and therapies for HIV/AIDS, malaria, TB, and NIDs must go together with capacity building, local empowerment, and social awareness.

## Supporting Information

Alternative Language Abstract S1 Translation of the Author Summary into Arabic by Marita Troye-Blomberg
(0.04 MB DOC)

Alternative Language Abstract S2 Translation of the Author Summary into Danish by T. Mark Doherty
(0.03 MB DOC)

Alternative Language Abstract S3 Translation of the Author Summary into French by Marita Troye-Blomberg
(0.03 MB DOC)

Alternative Language Abstract S4 Translation of the Author Summary into German by Andreas Thiel
(0.03 MB DOC)

Alternative Language Abstract S5 Translation of the Author Summary into Hungarian by Tamás Laskay
(0.04 MB DOC)

Alternative Language Abstract S6 Translation of the Author Summary into Italian by Diana Boraschi
(0.03 MB DOC)

Alternative Language Abstract S7 Translation of the Author Summary into Portuguese by Marita Troye-Blomberg
(0.03 MB DOC)

Alternative Language Abstract S8 Translation of the Author Summary into Spanish by Marita Troye-Blomberg
(0.04 MB DOC)

Alternative Language Abstract S9 Translation of the Author Summary into Swahili by Thomas Kariuki
(0.03 MB DOC)

Alternative Language Abstract S10 Translation of the Author Summary into Swedish by Marita Troye-Blomberg
(0.04 MB DOC)

## References

[1] Editorial (2007). Remembering the neglected tropical diseases.. Lancet.

[2] Molyneux DH, Hotez PJ, Fenwick A (2005). “Rapid-impact interventions”: how a policy of integrated control for Africa's neglected tropical diseases could benefit the poor.. PLoS Med.

[3] Mathers CD, Ezzati M, Lopez AD (2007). Measuring the burden of neglected tropical diseases: the Global Burden of Disease framework.. PLoS Negl Tropical Dis.

[4] Hotez PJ, Molyneux DH, Fenwick A, Ottesen E, Sachs SE, Sachs JD (2006). Incorporating a rapid-impact package for neglected tropical diseases with programs fro HIV/AIDS, tuberculosis, and malaria.. PLoS Med.

[5] Hotez PJ, Molyneux DH, Stillwaggon E, Bentwich Z, Kumaresan J (2006). Neglected tropical diseases and HIV/AIDS.. Lancet.

[6] Council Decision 971/2006/CE of 19/12/2006 adopting a Specific Programme for Research, Technological Development and Demonstration: ‘Cooperation’ (2007–2013). Official Journal of the European Union, L 54 of 22 February 2007, p. 30

[7] TDR business plan 2008–2013. Geneva, UNICEF/UNDP/World Bank/WHO Special Programme for Research and Training in Tropical Diseases, 2007 (TDR/GEN/EN/07.1/Rev.1)

[8] Keen J, Serghides L, Ayi K, Patel SN, Ayisi J (2007). HIV impairs opsonic phagocytic clearance of pregnancy-associated malaria parasites.. PLoS Med.

[9] Hotez PJ, Molyneux DH, Fenwick A, Kumaresan J, Sachs SE (2007). Control of neglected tropical diseases.. N Engl J Med.

[10] van Riet E, Hartgers FC, Yazdanbakhsh M (2007). Chronic helminth infections induce immunomodulation: consequences and mechanisms.. Immunobiology.

[11] Elias D, Britton S, Kassu A, Akuffo H (2007). Chronic helminth infections may negatively influence immunity against tuberculosis and other diseases of public health importance.. Expert Rev Anti Infect Ther.

[12] Dybul M (2006). Neglected tropical diseases and HIV/AIDS.. Lancet.

[13] Epstein MA (1984). Burkitt's lymphoma: clues to the role of malaria.. Nature.

[14] Ramaswamy M, Geretti AM (2007). Interactions and management issues in HSV and HIV coinfection.. Expert Rev Anti Infect Ther.

[15] Prentice AM, Ghattas H, Doherty C, Cox SE (2007). Iron metabolism and malaria.. Food Nutr Bull.

[16] Reiche EM, Bonametti AM, Voltarelli JC, Morimoto HK, Watanabe MA (2007). Genetic polymorphisms in the chemokine and chemokine receptors: impact on clinical course and therapy of the human immunodeficiency virus type 1 infection (HIV-1).. Curr Med Chem.

[17] Mockenhaupt FP, Cramer JP, Hamann L, Stegemann MS, Eckert J (2006). Toll-like receptor (TLR) polymorphisms in African children: common TLR-4 variants predispose to severe malaria.. J Commun Dis.

[18] Ferwerda B, McCall MB, Alonso S, Giamarellos-Bourboulis EJ, Mouktaroudi M (2007). TLR4 polymorphisms, infectious diseases, and evolutionary pressure during migration of modern humans.. Proc Natl Acad Sci USA.

[19] Khor CC, Chapman SJ, Vannberg FO, Dunne A, Murphy C (2007). A Mal functional variant is associated with protection against invasive pneumococcal disease, bacteremia, malaria and tuberculosis.. Nat Genet.

[20] Ebensen T, Guzmán CA (2008). Immune modulators with defined molecular targets: cornerstone to optimize rational vaccine design.. Hum Vaccin.

