# Supplementary material for: Immunity against HIV/AIDS, Malaria, and Tuberculosis during Co-Infections with Neglected Infectious Diseases: Recommendations for the European Union Research Priorities
Source: PLoS Negl Trop Dis. 2008 Jun 25;2(6):e255. doi: 10.1371/journal.pntd.0000255 (PMC2427178; doi:10.1371/journal.pntd.0000255)
Supplement: Alternative Language Abstract S1 — Translation of the Author Summary into Arabic by Marita Troye-Blomberg (0.04 MB DOC) [file pntd.0000255.s001.doc]

**(Arabic)**

المنخفضة الدخل ، لا سيما في افريقيا جنوب الصحراء الكبرى. الجزء الأكبر من اهتمام الرأي العام حتى الآن يسلط الضوء علي الثلاثة امراض، المدمرة ، فيروس نقص المناعة المكتسبة/ الايدز (HIV/ AIDS) والملاريا(Malaria) والدرن/ السل (tuberculosis, TB). ولكن ، في المناطق الريفية والمناطق الحضرية الفقيرة من البلدان المنخفضة الدخل ، فان عدد من الامراض المعدية المهملة تسبب معاناة هائلةكما انها تتلقى اهتماماً قليلاًَ او منعدماً الوسائط العلمية و الاعلامية. من خلال النظر في جميع الامراض المعدية المهملة (neglected infectious diseases NIDs) ، ويتضح انها تهدد صحة الافراد في البلدان الاقل نمواً الي حد مماثل كتهديد الامراض الرئيسية الثلاثة التي سبق ذكرها.

الجدير بالذكر ان مجموعة من ثلاثة عشر مرض من الامراض المعدية المهملة ، تشمل قرحة بورولي (*Mycobacterium ulcerae*)، والكوليرا (*Vibrio cholerae*) ، الدودة الغينية (*Guinea worm*) ، الاصابات بديدان التيرماتودا (trematodal infections) ، داء المذنبات الملتحية (hydatidosis) ، الليشمانيا (leishmaniasis) ، وداء الخيطيات اللمفاوية او داء الفيل (lymphatic filariasis, elephantiasis)، عمى الانهار(onchocerciasis) (river blindness) ، والبلهارسيا (schistosomiasis) ، داء الشريطية (helminthiasis)، التراكوما (chlamidia trachomatis) وداء التربانسوما او مرض النوم الافريقي (African sleeping sickness, Chagas disease) ، تصيب اكثر من مليار شخص (حوالي سدس سكان العالم). وبالنسبة لمعظم هذه الامراض ، فان اما اللقاحات غير متاحة ، اوغير فعالة ، أو باهظة الثمن. وعلاوة على ذلك ، كما ان غالبا الامراض المعدية المهملة (NIDs) تحدث بالمصاحبة بفيروس نقص المناعة المكتسبة/ الايدز، الملاريا او الدرن/ السل ، مشيرا الى ان الاصابة غالباً ما تكون مصحبة بالامراض المذكورة اعلاه هي القاعدة وليس الاستثناء في كثير من مناطق جغرافيه. هذا هو عنصر اساسي من اجل تطوير فعالية اللقاح واستراتيجيات العلاج، لا بد من فهم كيفية تحقيق المناعة لأحد مسببات مرض في الاشخاص ذوي الامراض متعددة. ومن بين العديد من برامج البحوث المتخصصة التي اطلقتها العديد من المنظمات الوطنية والدولية لفهم ومواجهة عبء فيروس نقص المناعة المكتسبة / الايدز والملاريا والسل ، وقليل تم إنجازه على وجه التحديد لمعالجة هذه المسألة المعقدة للمناعة من للامراض المصاحبة بواحد او اكثر من الثلاثة امراض الرئيسية القاتلة و الامراض المعدية المهملة (NIDs).

حتى الان ، فان المفوضية الأوروبية (European Commission) هي التي اقرت بضرورة اتباع سياسة نشطة لتطوير البحوث الجديدة او المحسنة والعلاجات الوقائية للامراض المعدية، بما فيها الادوية واللقاحات الجديدة للامراض المعدية المهملة. والمحافظة علي هذه البحوث في الاستمرارفي مواجهة تناقص الدعم الوطني. وفي حين ان البرنامج الاطاري السادس (6^th^ Framework Programme for European Commission ) الصادر عن المفوضية الأوروبية ، وكان معنونا أساسا للبحث المتعلق بمعالجة فيروس نقص المناعة المكتسبة/ الايدز والملاريا والسل. فان إطار البرنامج السابع (7^th^ Framework Programme for European Commission 2007-2013) سيشمل أيضا الامراض المعدية المهملة (NIDs). الالتزام الجديد في إطار البرنامج السابع يخلق فرصة لم يسبق لها مثيل الى العمل بنشاط على معالجة التحديات العلمية المرتبطة بالامراض المصاحبة و بين العدوى بفيروس نقص المناعة المكتسبة / الايدز والملاريا والسل ، الامراض المعدية المهملة (NIDs). وبالاضافة الى ذلك ، فان البرنامج الخاص للبحوث والتدريب في مجال امراض المناطق الحارة الصادر عن منظمة الصحة العالمية (WHO/ TDR) اظهر الاهتمام المتجدد لدعم البحوث بشأن الامراض المعدية المهملة (NIDs) لتشجيع الابتكار وتطوير المنتجات ، والتدخلات من اجل الوصول الى افضل النتائج. ومن المؤمل ان الصناعات العاملة في تطوير العقاقير واللقاحات سوف تعرب عن استراتيجيات جديدة حماية من الامراض المعدية المهملة (NIDs) في التقريرالصادر عن الاتحاد الأوروبي (WHO/TDR)، ولا سيما في مجال الحمايه المناعيه التي ترتبط بها ، وتطوير لقاحاتها.

لاعلان اهمية التعاون للتصدي للامراض المعدية المهملة (NIDs)، قام باحثون يمثلون اربعة عشر بلدا من افريقيا واوروبا واجتمعوا في اديس ابابا (اثيوبيا) 9-11 ايلول / سبتمبر ، 2007 ، لتحديد اولويات مشتركة لبحث الثغرات العلمية في هذا المجال. وقد عقد هذا الاجتماع من قبل اثنين من البرامج التابعة للمفوضية الاوروبية الممولة المبادرات الجارية ، وهما مشروع متكامل (MUVAPRED) وشبكة الامتياز (BIOMALPAR) ، وجمع على مستوى عال من الباحثين ، والأطباء ، وذوي الخبرة الصناعية ، فضلا عن ممثلين من المفوضية الاوروبية ومنظمة الصحة العالمية / البرنامج الخاص للبحوث والتدريب في مجال امراض المناطق الحارة. هذا التقرير يلخص اراء فريق الخبراء ، الذي اتخذ اسم AFRIEND (الشراكة الاوروبية - الافريقية للاهمال الامراض المعديةAFRIcan-European partnership for Neglected infectious Diseases ). ومن المتصور ان تعزز هذه الوثيقة النقاش في الأوساط العلمية ، وتقدم التوصيات بشأن الاجراءات التي ستتخذ في المستقبل من جانب المفوضية الاوروبية ومنظمة الصحة العالمية /البرنامج الخاص للبحوث والتدريب في مجال امراض المناطق الحارة و مجال الامراض المعدية المهملة والامراض المصاحبة.
