# Supplementary material for: Immunity against HIV/AIDS, Malaria, and Tuberculosis during Co-Infections with Neglected Infectious Diseases: Recommendations for the European Union Research Priorities
Source: PLoS Negl Trop Dis. 2008 Jun 25;2(6):e255. doi: 10.1371/journal.pntd.0000255 (PMC2427178; doi:10.1371/journal.pntd.0000255)
Supplement: Alternative Language Abstract S2 — Translation of the Author Summary into Danish by T. Mark Doherty (0.03 MB DOC) [file pntd.0000255.s002.doc]

# (Danish)

Infektionssygdomme forbliver en store sundhed og socioøkonomisk problem i mange lav-indkomst lande, specielt i sub-Sahara Afrika. Mest af offentlig opmærksomheden er hidtil blevet brugte på de tre mest knusende sygdomme, HIV/AIDS (human immundefekt virus), malaria, og tuberkulose (TB). Imidlertid i landlig og forarmede byområder af lav-indkomst lande, et antal forsømte infektionssygdomme (på engelske neglected infectious diseases eller NIDs) forårsager massive lidelser selvom de får lille eller ingen videnskabelig og masse-medier opmærksomhed. Ved at overveje alle NIDs sammen, det er åbenlyst at de truer sundheden af den ringeste til en lignende grade som de tre største sygdomme. Det antages at en gruppe af 13 forsømte infektionssygdomme, som inkludere Buruli sår (*Mycobacterium ulcerae*), kolera (*Vibrio cholerae*), cysticercosis, dracunculiasis (Guinea orm), trematoder, hydatidosis, leishmaniasis, lymfatic filariasis (elefantiasis eller Tropisk  trådormesygdom), onchocerkiasis, schistosomiasis (sneglefeber), helminthiasis, trachoma (*Chlamidia trachomatis*) and trypanosomiasis (Sovesyge) truer mere end et milliard mennesker (som korrespondere til 1/6 af jordens befolkning). For de meste af disse sygdomme, findes der ingen effektiv vacciner og dem som findes er for dyre. Desuden NIDs smitter også dem som allerede har HIV, malaria eller TB, som viser at infektion med flere patogene er reglen, ikke undtagelsen i mange geografiske områder. Dette er et nøglepunkt og hvis vi vil laver effektiv vaccine strategier er det altafgørende at vi forstår hvordan beskyttende immunitet kan være aktiveret i mennesker inficeret med flere patogene.

Blandt de mange forskning programmer lancerede af national og internationale organisationer til at forstår og konfrontere HIV, malaria og TB er der kun få som specifik adressere kompleks problemet af infektion med de tre dræber-sygdomme og NIDs på samme tid. Europa-kommisionen har anerkendt vigtighed af en aktiv forskning politik for udvikling af nye profylaktisk- og behandlings-midler for infektionssygdomme. For de meste, den Sjette Rammeprogram (FP6) fokuserede på forskning, der imødekommede udvikling i HIV, malaria og TB men den nye Syende Rammeprogram (FP7, 2007-2013) vil også inkludere NIDs. Den ny fokus på NIDs i FP7 giver et meget vigtigt lejlighed til at adressere de videnskabelige problemer som kommer fra infektion på samme tid med HIV, malaria eller TB og NIDs. Desuden den Special Programme for Research and Training in Tropical Diseases fra den Verdenssundhed Organisation (WHO/TDR) har vist fornyede interesse i praktisk forskning i NIDs. Den fornyelige opdateret strategi fra WHO/TDR har målet af mere støtte for forskning om forsømte infektionssygdomme ved at udvikle innovativ produkter og bedre adgang til behandling.

Til at adressere den voksende vigtighed af samtidigt infektioner, forskere fra 14 lande i Afrika og Europa mødt i Addis Ababa i Etiopien den 9 – 11 september 2007 til at identificere og prioritisere manglende områder for forsking. Mødet var kaldt af to omgående projekter støttede af Europa-kommisionen navnlig den MUVAPRED integrerede projekt og den BIOMALPAR netværk og samlede berømte klinisk forskere og industriforskere og deltager fra den Europa-kommisionen og WHO/TDR. Denne rapport præsenterer lighed fra af ekspertgruppen som var døbt AFRIEND (AFRIcan-European partnership for Neglected infectious Diseases). Vi håber at den dokument kunne provokere en debat i den videnskabelig kommunitet og aflever anbefalinger om aktioner i fremtiden af Europa-kommisionen og WHO/TDR i emnet af samtidigt infektioner og NIDs.
