# Supplementary material for: Immunity against HIV/AIDS, Malaria, and Tuberculosis during Co-Infections with Neglected Infectious Diseases: Recommendations for the European Union Research Priorities
Source: PLoS Negl Trop Dis. 2008 Jun 25;2(6):e255. doi: 10.1371/journal.pntd.0000255 (PMC2427178; doi:10.1371/journal.pntd.0000255)
Supplement: Alternative Language Abstract S3 — Translation of the Author Summary into French by Marita Troye-Blomberg (0.03 MB DOC) [file pntd.0000255.s003.doc]

**(French)**

Les maladies infectieuses restent un problème majeur socio-économique et de santé publique dans beaucoup de pays à faible revenu, plus particulièrement en Afrique subsaharienne. L’attention de la communauté internationale et du public est surtout portée sur les trois plus grandes maladies dévastatrices qui sont le VIH/SIDA, le paludisme et la tuberculose. Cependant, en milieu rural et semi urbain des pays à faible revenu un nombre de maladies infectieuses négligées cause d’importante souffrance bien que ceux-ci n’attirent l’attention de la communauté scientifique et des mass media. En considérant l’ensemble de ces maladies infectieuses négligées, il est évident qu’elles menacent la santé de ces couches vulnérables comme les trois plus grandes maladies infectieuses. Les données ont montré que les treize principales maladies infectieuses négligées, qui sont: l’ulcère de Buruli, le cholera, la cysticercose, la dracunculose, les trematodoses, les kystes hydatiques, l’onchocercose, les schistosomiases, les leishmanioses, les filarioses lymphatiques et les trypanosomiases (maladie du sommeil et de chagas) affectent plus d’un milliard de personnes soit le 1/6^eme^ de la population mondiale. Pour la plupart de ces maladies, les vaccins ne sont pas disponibles ou sont peu efficaces et ou sont très chers s’ils existent. Le plus souvent, ces maladies infectieuses négligées sont associées avec le VIH/SIDA, le paludisme ou la tuberculose, ce qui indique que les co-infections sont de règle dans les zones géographiques où ces maladies sont endémiques. Pour développer un vaccin efficace et des stratégies de traitement efficient, il est essentiel de comprendre comment l’immunité protectrice contre le pathogène pourrait être acquise chez les sujets infectés par plusieurs pathogenes.

Malgré les multiples efforts de recherche des groupes nationaux et internationaux pour comprendre et combattre les fardeaux du VIH/SIDA, le paludisme et de la tuberculose, peu de travaux sont réalisés dans le domaine complexe qui s’adresse au développement de l’immunité au cours des co-infections des trois grandes maladies infectieuses et les maladies infectieuses négligées. La Commission Européenne (CE) a reconnu la nécessité d’agir par une politique de la recherche active pour le développement de nouvelles stratégies pour améliorer les interventions préventives et de traitement des maladies infectieuses. Pendant que le 6^eme^ programme cadre de la CE était axé sur la recherche translationnel sur le VIH, le paludisme et la tuberculose, le 7^eme^ programme inclura aussi les maladies infectieuses négligées. Le nouvel engagement aux maladies infectieuses négligées dans le 7^eme^ Programme Cadre de la CE crée une occasion sans précédent, à s'occuper activement des défis scientifiques associés aux co-infections entre le VIH/SIDA, le paludisme, la tuberculose et les maladies infectieuses négligées. En outre, le programme spécial de Recherche et de formation sur les maladies infectieuses de l’OMS (WHO/TDR) a montré un regain d'intérêt pour la recherche translationnelle dans les maladies infectieuses négligées. Récemment mis à jour la stratégie de l’OMS/TDR vise à soutenir la recherche sur les maladies négligées en favorisant l'innovation pour le développement de produits, ainsi que pour l'accès à l'intervention.

Pour répondre à l'importance croissante de la co-infection, les scientifiques de 14 pays d'Afrique et d'Europe se sont réunis à Adis Abeba le 11 septembre 2007, en vue d'identifier et d’ hiérarchiser les lacunes dans ce domaine. La réunion a été convoquée par deux initiatives en cours financés par la CE, à savoir les projets MUVAPRED et le réseau d'excellence de BIOMALPAR et a regroupé de haut niveau d’experts scientifiques, des cliniciens, des industriels ainsi que des représentants de la CE et de L’OMS/TDR. Ce rapport résume le consensus du groupe d’experts qui a pris le nom de AFRIEND (AFRIcan-European partnership for Neglected infectious Diseases). Il est prévu que ce document pourrait favoriser un débat dans la communauté scientifique et de fournir des recommandations sur les activités futures de la CE et de l’OMS/TDR dans le domaine des co-infections et les maladies infectieuses négligées.
