# Supplementary material for: Immunity against HIV/AIDS, Malaria, and Tuberculosis during Co-Infections with Neglected Infectious Diseases: Recommendations for the European Union Research Priorities
Source: PLoS Negl Trop Dis. 2008 Jun 25;2(6):e255. doi: 10.1371/journal.pntd.0000255 (PMC2427178; doi:10.1371/journal.pntd.0000255)
Supplement: Alternative Language Abstract S4 — Translation of the Author Summary into German by Andreas Thiel (0.03 MB DOC) [file pntd.0000255.s004.doc]

# (German)

In vielen Entwicklungsländern und insbesondere im Afrika südlich der Sahara, sind Infektionskrankheiten immer noch ein Hauptproblem für Gesundheitspolitik und Sozioökonomie. Dabei richtete sich die öffentliche Aufmerksamkeit bisher vor allem auf verheerende Krankheiten wie HIV/AIDS, Malaria und Tuberkulose (TB). Gleichwohl verursachen weniger beachtete Infektionskrankheiten (neglected infectious diseases = NID) nicht minder immense Probleme und stehen dabei kaum im wissenschaftlichen oder öffentlichen Fokus. Zu den 13 häufigsten NIDs zählen Infektionskrankheiten wie das Buruli-Ulkus (*Mycobacteriu*m *ulcerae*), die Cholera (*Vibrio cholerae*), Zystizerkosen, die Drakunkulose (Guineawurm-Krankheit), Trematodeninfektionen, Echinokokkosen, Leishmanieninfektionen, die lymphatische Filariasis (*Elefantiasis*), die Onchozerkose (Flußblindheit), die Bilharziose, Helmintheninfektionen, Infektionen mit *Chlamydia trachomatis* und Trypanosomeninfektionen. Weit mehr als 1 Milliarde Menschen und damit 1/6 der Weltbevölkerung sind von diesen Krankheiten betroffen. Trotzdem sind für die meisten dieser Erkrankungen entweder keine oder nur wenig effiziente Impfstoffe vorhanden, die zudem meist häufig sehr teuer sind. Zudem Zudem treffen NIDs häufig mit HIV/AIDS, Malaria oder TB infizierte Individuen. Daher ist es von großer Wichtigkeit zu untersuchen, wie protektive Immunität gegenüber einem Pathogen bei gleichzeitiger Ko-Infektion mit multiplen weiteren Erregern induziert werden kann, um darauf aufbauend, effektive Impf- und Behandlungsstrategien zu entwickeln.

Unter den vielen aktuellen Förderprogrammen nationaler und internationaler Organisationen, die es sich es sich zum Ziel gesetzt haben, die Ausbreitung von HIV/AIDS, Malaria und TB zu stoppen, beschäftigen sich nur wenige Initiativen mit der Komplexität von Ko-Infektionen von HIV/AIDS, Malaria und TB anderen Infektionskrankheiten. Diese Problematik ist von der Europäischen Kommission (EC) erkannt worden, weshalb die Förderinitiativen im Bezug auf bessere Prophylaxe und Therapien in dieser Richtung verstärkt wurden. Während im 6. Rahmenprogramm (FP6) der EC hauptsächlich translationelle Projekte die sich mit HIV/AIDS, Malaria und TB beschäftigen, gefördert wurden, sollen innerhalb des 7. Rahmenprogramms (FP7, 2007-2013) auch Projekte zu NIDs unterstützt werden. Ein derartiges Engagement schafft großes Potezial, erstmals aktiv wissenschaftliche Problemstellungen im Bereich von HIV/AIDS, Malaria, TB und NIDs auch multidisziplinär zu bearbeiten. Zusätzlich hat jetzt auch das „*Special Programme for Research and Training in Tropical Diseases of WHO“* (WHO/TDR) sein Interesse an translationaler Forschung im Bereich der NIDs demonstriert. Das WHO/TDR will nun Innovationen im Hinblick auf neue Produkte fördern und so Forschung in bislang vernachlässigten Bereichen unterstützen.

Um die wachsende Bedeutung von Ko-Infektionen der Öffentlichkeit vorzustellen und um wissenschaftliche Schwerpunkte zu identifizieren und Engpässe aufzudecken, trafen sich Wissenschaftler aus 14 verschiedenen Ländern Europas und Afrikas im September 2007 in Addis Abeba (Äthiopien). An dem Treffen, das von MUVAPRED und BIOMALPAR, zwei Europäischen Forschungsnetzwerken initiiert wurde, nahmen Wissenschaftler, Kliniker, Firmenvertreter und Mitarbeiter der EC und des WHO/TDR teil. Dieser Artikel soll den Konsens der Expertengruppe zusammenfassen, die sich unter dem Namen AFRIEND (AFRIcan-European partnership for Neglected infectious Diseases) gegründet hat. sZiel dieses Dokumentes ist es zum einen eine Debatte in der wissenschaftlichen Gemeinschaft zu initiieren und zum anderen mögliche Anstöße bezüglich zukünftiger Aktionen der EC und des WHO/TDR zu geben.
