# Supplementary material for: Immunity against HIV/AIDS, Malaria, and Tuberculosis during Co-Infections with Neglected Infectious Diseases: Recommendations for the European Union Research Priorities
Source: PLoS Negl Trop Dis. 2008 Jun 25;2(6):e255. doi: 10.1371/journal.pntd.0000255 (PMC2427178; doi:10.1371/journal.pntd.0000255)
Supplement: Alternative Language Abstract S5 — Translation of the Author Summary into Hungarian by Tamás Laskay (0.04 MB DOC) [file pntd.0000255.s005.doc]

**(Hungarian)**

A fertőző betegségek még napjainkban is jelentős egészségügyi és szociális/gazdasági problémát jelentenek a szegényebb országokban, különösen a Szaharától délre fekvő afrikai államokban. A nyilvánosság figyelme eddig a három legtöbb áldozatot követelő fertőző betegségre, az AIDS-re (HIV fertőzés), a maláriára és a tuberkulózisra irányult. Mindazonáltal az alacsony jövedelmű országok városoktól távol eső és elszegényedett városi vidékein számos más, a közfigyelmet eddig elkerülő fertőző betegség, ún. elhanyagolt fertőző betegség (NIDs = neglected infectious diseases) is szedi áldozatait, melyek a tudományos kutatás és a tömegtájékoztató médiák érdeklődését szinte teljes mértékben nélkülözték. Pedig az NID csoportba tartozó betegségek együttesen legalább annyira fenyegetik a legszegényebb népesség egészségét, mint a három “fontos” fertőző betegség, a HIV/AIDS, a malária és a tuberkulózis. Számítások szerint a NID csoportba sorolt 13 betegség, a Buruli fekély (*Mycobacterium ulcerae*), a kolera (*Vibrio cholerae*), a ciszticerkózis, a dracunculiasis (*medinai féreg/Dracunculus medinensis*), a trematodák (szívóférgek) okozta fertőzések, a hidatidózis, a Leishmania fertőzés, az elefantiázis (nyirokúti fonálféreg fertőzés), az onchocerciasis (folyami vakság), a Schistosoma fertőzés, a bélférgesség (helminthiasis), a trachoma (*Chlamidia trachomatis*) és a Trypanosoma fertőzések (afrikai álomkór, Chagas-kór) több, mint egymilliárd embert, tehát a föld népességének egy hatodát veszélyeztetik. E fertőző betegségek legtöbbje ellen nincs védőoltás, vagy ha mégis van, az nem kellően hatékony vagy túl drága. Mindezeken felül a NID csoportba sorolt betegségek sokszor olyan betegeknél lépnek fel, és a világ sok területén ez inkább tűnik szabálynak, mint kivételnek, akik egyidejűleg HIV fertőzésben, maláriában vagy tuberkulózisban is szenvednek. Hatékony oltóanyagok és terápiás módszerek kifejlesztése szempontjából elengedhetetlen annak a megértése, hogyan lehet hatásos immunválaszt kiváltani olyan betegekben, akik egyszerre több fertőző betegségben szenvednek.

Habár egyes országok és nemzetközi szervezetek számos célirányos kutatási programot hívtak életre a HIV/AIDS, a malária valamint a tuberkulózis kutatására és leküzdésére, eddig kevés figyelem irányult az immunválasz vizsgálatára azokban a NID csoportba tartozó betegségekben, amelyek a három “fontos” fertőző betegséggel egyidejűleg fordulnak elő a betegekben. Az Európai Bizottság (EB) felismerte, hogy új és hatékonyabb megelőző és terápiás eljárások kifejlesztését célzó aktív kutatási politikára van szükség. Amíg azonban az EB 6. Keretprogramja (FP6) főleg a HIV/AIDS-szel, a maláriával és a tuberkulózissal kapcsolatos alkalmazott kutatásokra helyezte a fő hangsúlyt, az új 7. Keretprogramba (FP7, 2007-2013) az ún. elhanyagolt fertőző betegségek (NIDs) is bekerültek. A 7. Keretprogram ilyen irányú elkötelezettsége egyedülálló lehetőséget nyújt arra, hogy figyelmünket a NID betegségeknek a három “fontos” fertőző betegséggel (HIV/AIDS, malária, tuberkulózis) való együttes előfordulásából eredő tudományos kihívások felé fordítsuk. Az Egészségügyi Világszervezet (WHO) trópusi betegségekkel foglalkozó speciális kutatási és képzési programja (WHO/TDR) is megélénkülő érdeklődést mutat a NID betegségekkel kapcsolatos alkalmazott kutatások iránt. A WHO/TDR újonnan átdolgozott stratégiájának célja az eddig elhanyagolt kutatási területek, a termékfejlesztés és a betegségek leküzdésére irányuló intervenciós lehetőségekhez való hozzáférés támogatása.

A több fertőző betegség együttes fellépésének megvitatására 14 európai és afrikai ország tudósai gyűltek össze az etiópiai Addisz Abebában 2007. szeptember 9. és 11. között, hogy közösen megjelöljék, és fontosságuk szerint rangsorolják a többszörös fertőzések kutatása terén észlelhető hiányosságokat. A találkozón, amely az EB által támogatott két folyamatban levő kutatási program, a MUVAPRED és BIOMALPAR szervezésében jött létre, elismert tudományos kutatók, orvosok és ipari szakemberek, valamint az EB és a WHO képviselői vettek részt. Az alábbi cikkben összefoglaljuk a szakértők közös megegyezésen alapuló véleményét. További tevékenységéhez a szakértői csoport az AFRIEND (AFRIcan-European partnership for Neglected infectious Diseases) nevet választotta. Célunk, hogy ez a dokumentum a tudományos körökben vitára ösztönözzön. A dokumentum emellett ajánlásokat tartalmaz az EB és a WHO felé a társfertőzésekkel és az elhanyagolt fertőző betegségekkel (NID) kapcsolatos jövőbeli tevékenységükhöz.
