# Supplementary material for: Immunity against HIV/AIDS, Malaria, and Tuberculosis during Co-Infections with Neglected Infectious Diseases: Recommendations for the European Union Research Priorities
Source: PLoS Negl Trop Dis. 2008 Jun 25;2(6):e255. doi: 10.1371/journal.pntd.0000255 (PMC2427178; doi:10.1371/journal.pntd.0000255)
Supplement: Alternative Language Abstract S6 — Translation of the Author Summary into Italian by Diana Boraschi (0.03 MB DOC) [file pntd.0000255.s006.doc]

# (Italian)

Le malattie infettive costituiscono un problema sanitario e socio-economico sempre di grande importanza per molti paesi a basso reddito, in particolare nell’Africa sub-sahariana. L’attenzione pubblica finora è stata concentrata sulle tre malattie più devastanti, i “major killers” HIV/AIDS, malaria e tubercolosi (TB). Le aree rurali e quelle urbane più impoverite di moltissimi paesi a basso reddito sono però funestate da molte altre malattie infettive “dimenticate” (Neglected Infectious Diseases, NID) che causano grandi sofferenze ma che non attraggono l’attenzione pubblica né quella della comunità scientifica. Considerando tutte le NID, risulta chiaro che il rischio da esse posto alla salute delle popolazioni più povere è almeno pari a quello causato dai tre major killers. E’ stato calcolato che un gruppo di 13 NID, che include ulcera di Buruli (*Mycobacterium ulcerae*), colera (*Vibrio cholerae*), cisticercosi, dracunculiasi (verme della Guinea), infezioni da trematodi, idatidosi, leishmaniasi, filariasi linfatica (elefantiasi), oncocerchiasi (cecità dei fiumi), schistosomiasi, elmintiasi, tracoma (*Chlamidia trachomatis*) e tripanosomiasi (malattia del sonno, malattia di Chagas), colpisce oltre un miliardo di persone, cioè un sesto della popolazione mondiale. Per molte di queste malattie non esistono vaccini oppure i vaccini disponibili sono poco efficaci o troppo costosi. Si deve inoltre considerare che le NID colpiscono in genere persone già affette da HIV/AIDS, malaria o TB, e che la co-infezione è la regola piuttosto che l’eccezione nella gran parte dei paesi poveri. Dunque per poter sviluppare strategie di vaccinazione e di terapia veramente efficaci è essenziale capire come ottenere immunità protettiva in persone affette da infezioni concomitanti.

Nell’ambito dei molteplici programmi di ricerca lanciati da numerose organizzazioni nazionali e internazionali per affrontare il pericolo posto alla salute dell’umanità da HIV/AIDS, malaria e TB, pochissimo è stato fatto per esaminare in maniera mirata l’argomento assai complesso dell’immunità nelle situazioni di co-infezione fra i tre major killers e le NID. Finora è stata soprattutto la Commissione Europea (EC) a riconoscere la necessità di perseguire una politica attiva di ricerca per sviluppare o migliorare trattamenti profilattici e terapeutici per le malattie infettive. Mentre il Sesto Programma Quadro (FP6) di EC era diretto principalmente alla ricerca traslazionale per HIV/AIDS, malaria e TB, il nuovo Settimo Programma Quadro (FP7, 2007-2013) include anche le NID. Il nuovo impegno verso le NID in FP7 crea un’opportunità senza precedenti per affrontare attivamente la sfida scientifica posta dalle co-infezioni fra NID e i tre major killers HIV/AIDS, malaria e TB. Inoltre il Programma Speciale per la Ricerca e la Formazione per le Malattie Tropicali dell’Organizzazione Mondiale della Sanità (WHO/TDR) sta dimostrando un rinnovato interesse per la ricerca traslazionale nelle NID. La strategia di WHO/TDR, recentemente aggiornata, punta a sostenere la ricerca sulle malattie dimenticate sostenendo l’innovazione sia nello sviluppo di prodotti che per l’accesso ai trattamenti.

Per puntualizzare la crescente importanza dello studio delle co-infezioni, un gruppo di scienziati provenienti da 14 nazioni africane e europee si è riunito a Addis Ababa (Etiopia) dal 9 all’11 settembre 2007 con l’obiettivo di identificare e prioritizzare le necessità di ricerca nel campo delle co-infezioni. L’incontro è stato promosso congiuntamente da due iniziative europee già attive, il progetto integrato MUVAPRED e il network of excellence BIOMALPAR, e ha riunito scienziati, clinici ed esperti industriali di fama internazionale. Questo documento riassume le conclusioni del gruppo di esperti che ha preso il nome di AFRIEND (AFRIcan-European partnership for Neglected infectious Diseases). Si prevede che tale documento possa stimolare un fattivo dibattito nella comunità scientifica e fornire raccomandazioni per le azioni che EC e WHO/TDR intraprenderanno in futuro nel campo delle co-infezioni e delle NID.
