# Supplementary material for: Immunity against HIV/AIDS, Malaria, and Tuberculosis during Co-Infections with Neglected Infectious Diseases: Recommendations for the European Union Research Priorities
Source: PLoS Negl Trop Dis. 2008 Jun 25;2(6):e255. doi: 10.1371/journal.pntd.0000255 (PMC2427178; doi:10.1371/journal.pntd.0000255)
Supplement: Alternative Language Abstract S7 — Translation of the Author Summary into Portuguese by Marita Troye-Blomberg (0.03 MB DOC) [file pntd.0000255.s007.doc]

**(Portuguese)**

As doenças infecciosas permanecem um grande problema, a nível de saúde e socio-económico, em muitos países sub-desenvolvidos, particularmente a sul do Sahara em África. A maioria da atenção do público tem-se virado para as três doenças mais devastadoras, VIH/SIDA, malária e tuberculose. Contudo, em áreas rurais e em áreas urbanas mais empobrecidas de países sub-desenvolvidos, um número de doenças infecciosas negligenciadas (DINs) tem provocado um enorme sofrimento apesar de receberem pouca ou nenhuma atenção, quer científica, quer por parte da comunicação social. Considerando as DINs em conjunto, é óbvio que elas ameaçam a saúde dos mais necessitados com uma extensão semelhante aos três maiores assassinos. Foi calculado que um grupo de 13 DINs, incluindo a úlcera do Buruli (*Mycobacterium ulcerae*), cólera (*Vibrio cholerae*), cisticercose, dracunculiase (verme da Guiné), infecções por trematódeos, hidatidose, leishmaniose, filariose linfática (elefantíase), oncocercíase (cegueira dos rios), schistosomíase, helmintíase, tracoma (*Chlamidia trachomatis*) e tripanossomíase (doença do sono africana, doença das Chagas), afectam mais de um milhar de milhões de pessoas (o que corresponde a um sexto da população mundial). Para a maioria das doenças, as vacinas não estão disponíveis, ou são pouco eficazes, ou são muito caras. É ainda de referir que as DINs ocorrem com frequência em indivíduos que também se encontram afectados com HIV/SIDA, malária ou tuberculose, indicando que as co-infecções são a regra em vez da excepção em muitas áreas geográficas. Isto é um elemento chave e, para que seja desenvolvida uma vacina eficaz e estratégias para tratamentos, é essencial perceber como pode ser conseguida protecção imunitária contra um patogénio em pessoas co-infectadas com múltiplos patogénios.

Entre numerosos programas de investigação específicos lançados por muitas organizações nacionais e internacionais para perceber e confrontar as consequências do HIV/Sida, malária e tuberculose, pouco tem sido feito para determinar especificamente esta complexa questão da imunidade durante co-infecções com os três maiores assassinos e as DINs. A Comissão Europeia (CE) reconheceu a necessidade de prosseguir com uma política de investigação activa para melhorar ou desenvolver novos procedimentos profiláticos e tratamentos para doenças infecciosas. Enquanto que o 6º Programa quadro (6^th^ Framework Programme- FP6) da Comissão Europeia tem-se preocupado principalmente com investigação em HIV/SIDA, malária e tuberculose, o 7º Programa quadro (FP7, 2007-2013) irá também incluir as DINs. É de acrescentar que o Programa Especial de Investigação e Formação em Doenças Tropicais da WHO (Special Programme for Research and Training in Tropical Diseases of WHO - WHO/TDR) mostrou interesse renovado numa investigação contínua em DIN’s. A estratégia mais recente da WHO/TDR tem como objectivo suportar a investigação das necessidades negligenciadas, promovendo inovação no desenvolvimento de produtos e para acesso a intervenções.

Para determinar a importância cada vez maior das co-infecções, cientistas de 14 países diferentes em África e na Europa encontraram-se em Addis Ababa (Etiópia) em 9-11 de Setembro, 2007, para em conjunto identificar e tornar prioritárias as falhas na investigação nesta área. O encontro foi convocado por duas iniciativas fundadas pela CE, a MUVAPRED integrated project e a BIOMALPAR network of excellence, e juntaram cientistas de topo, clínicos, peritos na indústria assim como os representantes da CE e da WHO/TDR. Este relatório sumariza o consenso deste grupo perito que adoptou o nome de AFRIEND (AFRIcan-European partnership for Neglected infectious Diseases). Pensa-se que este documento possa promover um debate na comunidade científica e providenciar recomendações em acções futuras pela CE e a WHO/TDR na área das co-infecções e DINs.
