# Supplementary material for: Immunity against HIV/AIDS, Malaria, and Tuberculosis during Co-Infections with Neglected Infectious Diseases: Recommendations for the European Union Research Priorities
Source: PLoS Negl Trop Dis. 2008 Jun 25;2(6):e255. doi: 10.1371/journal.pntd.0000255 (PMC2427178; doi:10.1371/journal.pntd.0000255)
Supplement: Alternative Language Abstract S8 — Translation of the Author Summary into Spanish by Marita Troye-Blomberg (0.04 MB DOC) [file pntd.0000255.s008.doc]

**(Spanish)**

Las enfermedades infecciosas siguen siendo un importante problema socioeconómico y de salud en muchos países de ingreso bajo, particularmente en África al sur del Sahara. La mayoría de la atención pública se ha dedicado hasta ahora a las tres enfermedades más devastadoras, HIV/SIDA, malaria y tuberculosis (TB). Sin embargo, en áreas rurales o urbanas empobrecidas de países de ingreso bajo, varias enfermedades infecciosas ignoradas (NIDs abr.Neglected Infectious Diseases) causan sufrimientos masivos. Aun así estas enfermedades reciben poca o ninguna atención tanto científica como de los medios de comunicación. Considerando todas las NIDs juntas, es obvio que presentan una amenaza a la salud de los más pobres en un grado similar a los tres asesinos principales. Se ha calculado que un grupo de 13 enfermedades infecciosas ignoradas, incluyendo la úlcera de Buruli (*Mycobacterium ulcerae*), el cólera (*Vibrio cholerae*), la cysticercosis, la dracunculiasis (gusano de Guinea), infecciones con trematodes, hydatidosis, leishmaniasis, el filariasis linfático (elephantiasis), la onchocerciasis (ceguera del río), la schistosomiasis, la helmintiasis, el tracoma (*Chlamidia trachomatis*) y la trypanosomiasis (enfermedad del sueño africana, mal de Chagas) afecta a más de mil millones de personas (lo que corresponde a un sexto de la población mundial). Para la mayoría de estas enfermedades, las vacunas son poco eficaces, demasiado costosas o simplemente no existen. Por otra parte, las NIDs son frecuentes en los mismos individuos también afectados por el HIV/SIDA, malaria o TB, indicando que las co-infecciones son, más que la excepción, la regla en muchas áreas geográficas. Esto es un aspecto clave, puesto que para desarrollar estrategias eficaces de vacunación y tratamiento, es esencial entender cómo la inmunidad protectora se puede alcanzar en pacientes co-infectados con múltiples patógenos.

Entre los numerosos programas de investigación lanzados por varias organizaciones, nacionales e internacionales, con el fin de entender y enfrentar los problemas ocasionados por HIV/SIDA, malaria y TB, poco se ha hecho para tratar específicamente el asunto complejo de la inmunidad durante co-infecciones entre los tres asesinos y las principales NIDs. La Comisión de la Unión Europea (UE) ha reconocido la necesidad de desarollar activamente una política de investigación con el fin de conseguir nuevas o mejoradas medidas profilácticas y tratamientos para las enfermedades infecciosas. Mientras que el Sexto Programa Marco para la investigación (FP6) de la UE trataba principalmente de la investigación de translación para HIV/SIDA, malaria y TB, el nuevo 7° programa (FP7, 2007-2013) también incluirá a las NIDs. La introduccion del estudio de las NIDs en FP7 crea una oportunidad sin precedente para tratar activamente los desafíos científicos asociados a co-infecciones entre HIV/SIDA, malaria, TB, y las NIDs. Además, el programa especial para la investigación y la educación en enfermedades tropicales “the Special Programme for Research and Training in Tropical Diseases of WHO” (WHO/TDR) ha demostrado renovado interés para la investigación de translación en NIDs. La estrategia recientemente actualizada de WHO/TDR tiene como objetivo apoyar la investigación relacionada con las necesidades ignoradas, de esta manera fomentando la innovación del desarrollo de producto, y del acceso a las intervenciones.

Para tratar el importante asunto de aumento de co-infecciones, expertos de 14 países distintos de África y Europa se reunieron en Addis Ababa (Etiopía) del 9-11 de Septiembre 2007, para conjuntamente identificar posibles huecos de conocimiento y dar prioridad a la investigación en estas áreas. Esta reunión fue convocada por dos iniciativas financiadas por la UE, el proyecto integrado MUVAPRED y la red de excelencia BIOMALPAR, donde se juntó a científicos de alto nivel, clínicos, tecnicos en maestría industrial así como representantes de la UE y WHO/TDR. Este informe resume el consenso del grupo de expertos, que tomó el nombre de AFRIEND (AFRIcan-European partnership for Neglected Diseases esp. sociedad Africano-Europea para las enfermedades infecciosas ignoradas). Se considera que este documento podría fomentar una discusión en la comunidad científica y proporcionar recomendaciones para las futuras acciones de la UE y el WHO/TDR en el área de co-infecciones y de las NIDs.
