# Supplementary material for: Immunity against HIV/AIDS, Malaria, and Tuberculosis during Co-Infections with Neglected Infectious Diseases: Recommendations for the European Union Research Priorities
Source: PLoS Negl Trop Dis. 2008 Jun 25;2(6):e255. doi: 10.1371/journal.pntd.0000255 (PMC2427178; doi:10.1371/journal.pntd.0000255)
Supplement: Alternative Language Abstract S9 — Translation of the Author Summary into Swahili by Thomas Kariuki (0.03 MB DOC) [file pntd.0000255.s009.doc]

**(Swahili)**

Marathi yanayo sababishwa na viini tofauti yaendelea kuathiri maisha na kudunisha hali ya uchumi wa wanaadamu wenye mapato ya chini hasa katika nchi za kusini mwa jangwa la Sahara. Magonjwa yanayo zingatiwa zaidi ni ukimwi, malaria na kifua kikuu “magonjwa matatu yenye athari sana”. Walakin, katika sehemu za mashambani na vitongoji duni wanamoishi watu wenye mapato madogo zinakumbwa na athari ya magonjwa mengi ambayo “yamepuuzwa” kwa muda mrefu. Hali hii imesababisha athari nyingi kwa binadamu katika sehemu mbalimbali za nchi zinazoendelea. Ukiangaziya magonjwa yote “yaliyopuuzwa” kwa jumla ni dhahiri yakwamba yana thoofisha afya sawa na yale “magonjwa matatu” kwa watu wenye mapato madogo. Imeokisiwa yakwamba kikundi cha magonjwa 13 kati ya yale magonjwa “yaliyopuuzwa” kama vile kidonda cha Buruli (*Mycobacterium ulcerae*), kipindupindu (*Vibrio cholerae*), minyoo ya mara, drakunkuliasis (Guinea worm), magonjwa yanayo sababishwa na minyoo, haidatikosis, leshmaniyasis, tezi (elephantiasis), minyoo ya upofu wa macho (river blindness), kichocho, minyoo ya tumbo, trachoma (*Chlamidia trachomatis*) na ugonjwa wa malale yana athiri watu zaidi ya billioni moja (kama asili mia sitini ya watu duniani). Marathi haya hayana kinga au chanjo haithabiti sawa, ama chanjo zilizopo ni bei ghali sana. Marathi haya humthuru zaidi mtu ambae ana ugonjwa wa ukimwi, malaria au kifuwa kikuu, kuonyesha yakwamba kupata magonjwa mawili kwa pamoja siku hizi ni kawaida wala sijambo la kushangaza katika sehemu nyingi tofauti duniani. Hili jambo ni muhimu kulitambua, na ili tuweze kubuni chanjo ama madawa ya kupambana na magonjwa mawili tofauti kwa pamoja, nimuhimu kuelewa vile mwili wa binaadamu unaweza kujikinga na viini vinavyo sababisha marathi kwenye mwili wa watu wenye magonjwa mawili au zaidi kwa pamoja.

Mipangingilio mengi ya utafiti yanayo nufaika kifedha kutoka kwa serekali ama kutoka kwa mashirika ya ulimwengu yamebuniwa hasa kuangazia athari zinazo letwa na ukimwi, malaria na kifua kikuu. Yamepuuza utafiti upande wa kuchunguza kinga za mwili wa mtu anapo uguwa kutokana na ugonjwa mmoja kati ya yale “makubwa matatu” pamoja na ugonjwa mmoja wa yale “yaliopuuzwa”. Mataifa ya muungano wa yuropa (EC) yameona umuhimu wa kuanzisha kampeni za kuweka misingi ya utafiti wa madawa ya kujikinga ama kuponya marathi haya yanayo ambukizwa na viini mbalimbali. Ambapo Mpangilio wa Sita (Framework Programme, FP6) wa EC uliangaziya sana utafsiri wa utafiti upande wa “magonjwa matatu muhimu”, Mpangilio mpya wa Saba (Framework Programme (FP7, 2007-2013) utajumlisha pia “magonjwa yaliopuuzwa”. Ukurasa huu mpya wakuangaliya umuhimu wa utafiti juu ya “magonjwa yaliyopuuzwa” katika ukurasa wa FP7 utazungumzia juu ya njia mpya za kuchunguza hali ya mwanadamu anapopata ugonjwa mmoja kati ya yale “magonjwa yalioyopuuzwa” pamoja na ugonjwa mmoja kati ya yale “magonjwa muhimu”. La ziada ni shirika la Ulimwengu la Afya (WHO/TDR) limeonyesha dalili za kuunga mkono harakati hizi za utafiti wa “magonjwa yaliopuuzwa”.

Kuagazia zaidi umuhimu wa utafiti wa magonjwa mawili tofauti kwa pamoja, watafiti kutoka nchi 14 za Africa na bara la Yuropa yalikutana Adis Ababa (Ethiopia) mnamo Septemba 9 hadi11, 2007, ili washirikiane pamoja kujua ni lipi linalo hitaji suluhisho la kitafiti kwa haraka katika kuangamiza magonjwa yaliopuuzwa yanapo mpata mtu ambaye tayari anaugua moja kati ya “magonjwa muhimu”. Mkutano huu ulidhaminiwa na miradi miwili ya EC, kwa majina ni mradi wa MUVAPRED na mtandao BIOMALPAR. Mkutano huu ulikutanisha watafiti wanaoongoza katika utafiti wa magonjwa haya, madaktari na wanaomiliki viwanda vya kutengeneza madawa pamoja na washiriki kutoka EC na WHO/TDR. Repoti hino imefupisha yaliyo zungumziwa na kukubaliwa na walioshiriki. Sasa kikundi hiki kinajiita AFRIEND (AFRIcan-European partnership for neglected Infectious Diseases). Inatarajiwa kwamba hii repoti itaanzisha mazungumzo kati ya watafiti wa magonjwa “yaliyopuuzwa” na wale wa “magonjwa muhimu matatu”. Mazungumzo hayo yanatarajiwa kutoa muwelekeo utakaochukuliwa na mashirika ya EC na WHO/TDR upande wa utafiti wa magonjwa “yaliyopuuzwa” yanapompata mtu ambaye ana moja kati ya “magonjwa muhimu matatu”.
