# Supplementary material for: Immunity against HIV/AIDS, Malaria, and Tuberculosis during Co-Infections with Neglected Infectious Diseases: Recommendations for the European Union Research Priorities
Source: PLoS Negl Trop Dis. 2008 Jun 25;2(6):e255. doi: 10.1371/journal.pntd.0000255 (PMC2427178; doi:10.1371/journal.pntd.0000255)
Supplement: Alternative Language Abstract S10 — Translation of the Author Summary into Swedish by Marita Troye-Blomberg (0.04 MB DOC) [file pntd.0000255.s010.doc]

# (Swedish)

Infektionssjukdomar är fortfarande ett stor hälso- och socioekonomiskt problem i många fattiga länder, speciellt i länderna söder om Sahara. Fram till nu, har världen koncentrerat sig på de tre mest förödande sjukdomarna, HIV/AIDS, malaria och tuberkulos (TB). Det är dock helt klart, att det ute på landsbygden och i vissa stadsregioner av fattiga länder, finns ett stort antal mer eller mindre bortglömda infektionssjukdomar, som allmänt kallas “neglected infectious diseases” (NIDs), som förorsakar mycket lidande, men som trots detta inte får någon uppmärksamhet, varken från forskare eller media. Om man tar i beräkning alla dessa NIDs, så är det helt klart att de är ett lika stort hot mot den fattiga befolkningen som de tre stora sjukdomarna. Man har beräknant att en grupp av 13 olika NIDs, inkluderande Buruli ulcer (*Mycabacterium ulcerae*), cholera (*Vibrio cholerae*), cysticercosis, dracunculiasis (Guine worm), trematod infektioner, hydatidosis, leishmania, trakom (*Chlamidia trachomatis*) och afrikansk sömnsjuka (Chagas), infekterar mer än en miljard människor, vilket motsvarar en sjättedel av världens befolkning, För de flesta av dessa sjukdomar finns det inga vacciner, eller vaccinerna ger dåligt skydd eller också är de för dyrbara för denna grupp av världens befolkning. Många av dessa NIDs finns hos människor som redan är smittade av HIV/AIDS, malaria eller TB, och därför är dubbelinfektioner mer är regel än undantag. Denna kunskap är mycket viktig eftersom detta kan påverka en individs skyddande immunitet och effektiviteten av vacciner. Det är alltså av stor vikt att förstå hur skyddande immunitet kan uppnås i individer som är smittade med en eller flera NIDs.

Bland alla de forskningsprogram som lanserats av många nationella och internationella organisationer, för att bättre förstå och minimera effekten av HIV/AIDS, malaria och TB har inte mycket gjorts för att specifikt studera hur dubbelinfektioner mellan de tre stora och NIDs påverkar värdens immunologiska svar. Den Europeiska Kommissionen (EC) har erkänt detta och förespråkar behovet av en aktiv forskningspolitik för att utveckla nya och förbättrade profylaktika och behandlingsmetoder mot dessa infektionssjukdomar. EU’s 6^e^ ramprogram var främst riktat mot behandlingmetoder av HIV/AIDS, malaria och TB, medan det 7^e^ ramprogrammet (FP7, 2007-2013) också kommer att inkludera NIDs. Detta nya åtagande inom FP7 kommer att ge en helt ny och unik möjlighet att studera den vetenskapliga utmaningen som dubbelinfektioner mellan HIV/AIDS, malaria, TB och NID’s innebär. På samma gång har världshälsoorganisationen för forskning och träning i tropiska sjukdomar (WHO/TDR) visat ett förnyat intresse för forskning inom NID’s. WHO/TDR’s nyligen uppdaterade strategidokument syftar till att stödja forskning som förväntas leda till nya innovativa produkter och förbättrade behandlingsmetoder mot NIDs.

För att bemöta vikten and dubbelinfektioner, träffades forskare från 14 olika länder i Afrika och Europa i Addis Ababa (Etiopien), den 9-11 september, 2007, för att gemensamt identifiera och prioritera forskningsgapen som finns inom detta område. Sammankallande för mötet var två pågående EU-stödda initiativ, nämligen det integrerade initiativet MUVAPRED och excellensnätverket BIOMALPAR som lyckades samla en grupp av excellenta forskare, kliniker och expertis från industrin så väl som representanter från EU och WHO/TDR. Denna rapport summerar den samlade opinionen av expertgruppen, som antog namnet AFRIEND (AFRIcan-European partnership for Neglected infectious Diseases). Vi hoppas att detta dokument skall bidraga till en debatt inom den vetenskapliga världen och ge rekommendationer till EU och WHO/TDR för framtida satsningar inom området dubbelinfektioner och NIDs.
